# Red-Shifted FRET Biosensors for High-Throughput Fluorescence Lifetime Screening

**DOI:** 10.3390/bios8040099

**Published:** 2018-10-24

**Authors:** Tory M. Schaaf, Ang Li, Benjamin D. Grant, Kurt Peterson, Samantha Yuen, Prachi Bawaskar, Evan Kleinboehl, Ji Li, David D. Thomas, Gregory D. Gillispie

**Affiliations:** 1 Department of Biochemistry, Molecular Biology and Biophysics, University of Minnesota, Minneapolis, MN 55455, USA; tms@ddt.umn.edu (T.M.S.); ang@ddt.umn.edu (A.L.); sly@ddt.umn.edu (S.Y.); prachi@ddt.umn.edu (P.B.); evan@ddt.umn.edu (E.K.); seekor@gmail.com (J.L.); 2 Fluorescence Innovations Inc., Minneapolis, MN 55455, USA; bdgrant12@gmail.com (B.D.G.); kurt.c.peterson@gmail.com (K.P.); 3 Photonic Pharma LLC, Minneapolis, MN 55410, USA

**Keywords:** time-resolved FRET, biosensor, drug screening, SERCA, small-molecule, high-throughput, plate reader, fluorescence, fluorescent proteins

## Abstract

We have developed fluorescence resonance energy transfer (FRET) biosensors with red-shifted fluorescent proteins (FP), yielding improved characteristics for time-resolved (lifetime) fluorescence measurements. In comparison to biosensors with green and red FRET pairs (GFP/RFP), FPs that emit at longer wavelengths (orange and maroon, OFP/MFP) increased the FRET efficiency, dynamic range, and signal-to-background of high-throughput screening (HTS). OFP and MFP were fused to specific sites on the human cardiac calcium pump (SERCA2a) for detection of structural changes due to small-molecule effectors. When coupled with a recently improved HTS fluorescence lifetime microplate reader, this red-shifted FRET biosensor enabled high-precision nanosecond-resolved fluorescence decay measurements from microliter sample volumes at three minute read times per 1536-well-plate. Pilot screens with a library of small-molecules demonstrate that the OFP/MFP FRET sensor substantially improves HTS assay quality. These high-content FRET methods detect minute FRET changes with high precision, as needed to elucidate novel structural mechanisms from small-molecule or peptide regulators discovered through our ongoing HTS efforts. FRET sensors that emit at longer wavelengths are highly attractive to the FRET biosensor community for drug discovery and structural interrogation of new therapeutic targets.

## 1. Introduction

Two-color fluorescent protein biosensors, expressed in living cells, offer a powerful approach for measuring molecular structural changes by fluorescence resonance energy transfer (FRET) [1] to evaluate biomolecular mechanisms and develop therapeutic approaches [2]. This is especially powerful when FRET is measured by a decrease in the donor’s fluorescence lifetime (FLT), increasing the precision of detecting protein structural changes by a factor of 30, compared with the measurement of FRET by intensity [3,4]. However, the throughput (rate at which successive measurements can be made at equivalent precision) for FLT detection is limited to about 0.1 per second for 1% precision, using the conventional method of time-correlated single-photon counting (TCSPC). Recently developed technology, using direct waveform recording (DWR), has increased the throughput of high-precision FLT detection by 5 orders of magnitude, to 10,000 per second [4]. DWR works by directly digitizing the entire waveform (thousands of photons) from a single intense laser pulse, using modern microchip lasers (extremely reproducible laser pulse shape) and a proprietary digitizer that yields extremely reproducible signals [4]. The DWR approach has enabled (a) the detection of protein structural changes with high precision during submillisecond biochemical transients [5] and (b) high-throughput screening (HTS) using the first truly high-throughput nanosecond-resolution fluorescence lifetime microplate reader (FLT-PR) [2,6,7]. In the FLT-PR, throughput is limited by the rate at which the plate can be translated, so the effective increase in throughput is ~100-fold, compared with TCSPC. The FLT-PR reads samples at the same rate (hundreds per minute) as a conventional intensity-based plate reader, increasing the precision of FRET detection in HTS by a factor of 30 [2,6,7].

This increased detection precision, coupled with the engineering of fluorescent biosensors in living cells, promises a revolution in the precision of drug discovery by HTS using large compound libraries. In our previous FLT studies in living cells, the target protein of interest was fused to green fluorescent protein (GFP, the FRET donor) and red fluorescent protein (RFP, the FRET acceptor), producing a two-color intramolecular biosensor [2]. In other cases, GFP and RFP were fused to separate interacting proteins, producing an intermolecular biosensor [8]. However, the reliability and sensitivity of this approach is limited by interference from light scattering, cellular autofluorescence, and the intrinsic fluorescence of the small molecules being tested [9,10]. In the present study, we show that these problems are greatly alleviated by using fluorescent proteins (FPs) that absorb and emit at longer wavelengths.

Fluorescence resonance energy transfer (FRET) biosensors engineered with red-shifted fluorescent proteins offer several potential advantages, in comparison to the more commonly used GFP and RFP [11]. The primary advantage is improved signal-to-background, as the relative contribution of interference from cellular autofluorescence or other sources is dramatically decreased. Further potential advantages include improved FP characteristics from reduced dimerization, longer Stokes shift, or improved FP maturation [12]. Fortunately, genetically-encoded red-shifted FRET biosensors, with excitation wavelengths greater than 530 nm, have recently been developed for live-cell applications [13,14]. In addition, these red-shifted biosensors typically have a longer fluorescence lifetime than that of GFP [15], which should improve the dynamic range of the biosensor. However, the improvement that this provides for FLT-based assays has yet to be described in detail, especially in HTS applications.

In the present study, we demonstrate the optimization of red-shifted FRET biosensors for FLT detection at HTS speeds, using a recently developed and refined high-throughput microplate reader capable of measuring the nanosecond FLT decay in a high-density microplate format (1536 wells, 5 μL/well), acquiring data from a full plate in less than three minutes with lifetime measurement precision (standard deviation/mean) of 1% or better. This remains the only instrument that can practically apply fluorescence lifetime technology to HTS.

**Figure A1:**
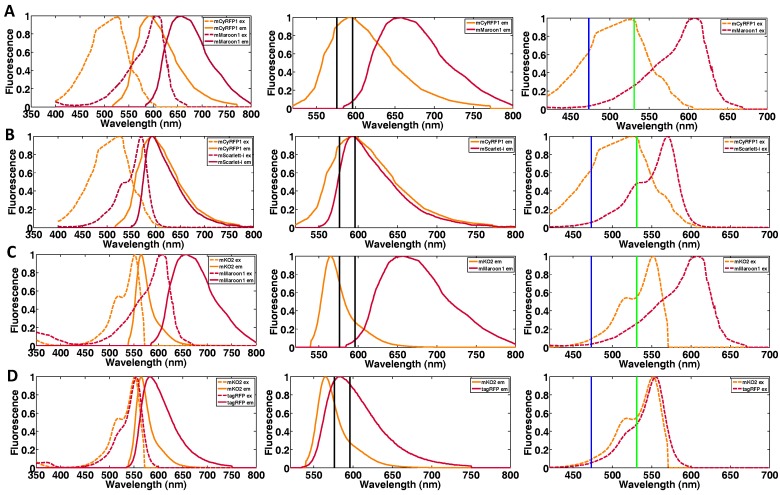
Red-shifted fluorescent protein FRET pairs. (**A**) mCyRFP1/mMaroon, (**B**) mCyRFP1/mScarlett-i, (**C**) mKO2/mMaroon, (**D**) mKO2/tagRFP. The excitation spectra and emission spectra of each donor and acceptor fluorescent protein are shown in right figure for each FRET pair. A 586/20 fluorescence emission band pass filter is shown between black bars in middle figure for each FRET pair. This band pass filter should be optimal for detection of the donor lifetime without interference from the acceptor. The left figure shown for each FRET pair depicts the excitation spectra with the blue line indicating 473 nm laser excitation and the green line 532 nm laser excitation.

We have found that the expression of genetically-encoded biosensors, fusing GFP and RFP to the target protein, not only provides the expected advantages of a live-cell assay, because compounds must permeate the cell under truly physiological conditions, but also improves the uniformity of the biosensor itself, because it eliminates the heterogeneity introduced by protein purification and labeling with fluorescent dyes [16,17]. Careful review of the currently available red-shifted FP pairs (Figure A1) allowed us to select appropriate donor and acceptor FPs to improve our biosensors for FLT detection. Fortunately, a new FRET pair was recently reported for fluorescence lifetime imaging (FLIM) [18]. This FRET pair consists of mCyRFP1/mMaroon1 (here designated OFP/MFP for simplicity).

**Figure 1:**
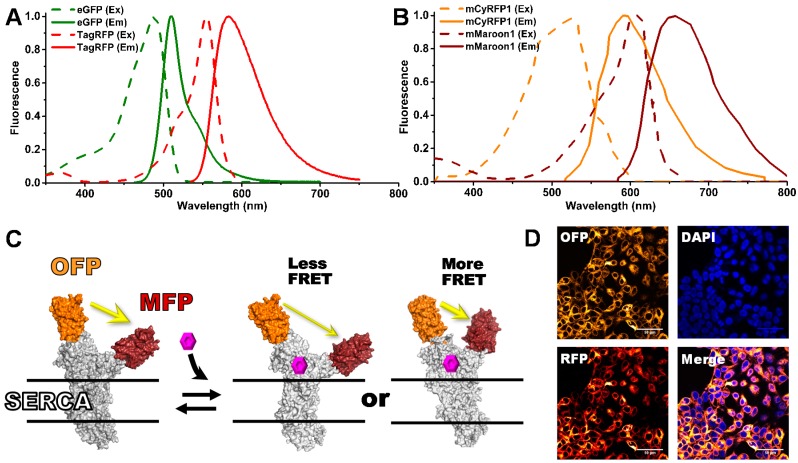
(**A**) Previously used fluorescence resonance energy transfer (FRET) pair eGFP/tagRFP (G/R) excitation (dashed) and emission (solid) spectra. (**B**) Red-shifted FRET pair mCyRFP/mMaroon (O/M) excitation and emission spectra. (**C**) The two-color SERCA (2CS) intramolecular FRET biosensor monitors the structural status of SERCA’s cytoplasmic domains, which may be affected by the binding of small molecules. (**D**) Confocal microscopy of HEK293 stable cell line expressing 2CS FRET biosensors.

To compare the performance of the two biosensors directly, we replaced GFP and RFP with OFP and MFP in our most thoroughly tested FRET biosensor, SERCA2a, the human cardiac calcium pump [1,19,20]. In this construct, expressed in HEK293 cells, the donor is inserted into an internal loop, and the acceptor is placed at the N terminus (Figure 1C). The data reported here shows that for the SERCA2a target, OFP/MFP nearly doubles the FRET signal window, due to the increased dynamic range of the OFP donor lifetime for a given drug-induced structural perturbation. This new FRET pair’s improved sensitivity is probably due largely to the improved overlap between the spectra of donor emission and acceptor absorbance, giving rise to a larger R_0_ value (distance for 50% FRET) and thus substantially greater sensitivity to structural changes in the target (Figure 1A,B). Subtle differences in the endogenous linkers and/or structures of the fluorescent proteins may alter chromophore orientation, which can also affect R_0_, and thus sensitivity. There are some minor disadvantages to these red-shifted biosensors: slightly lower molar absorptivity, greater photolability, and sensitivity to photobleaching [18], but we have found that these do not significantly impact the improved HTS quality in comparison to the GFP/RFP FRET biosensor. Just as the CFP/YFP FRET pair has been gradually replaced by the red-shifted GFP/RFP pair, we predict that OFP/MFP will become the future FRET pair of choice for live-cell applications.

## 2. Materials and Methods

### 2.1. Cell Culture and Stable Clone Generation

HEK293 (originally derived from human embryonic kidney) cells were maintained in phenol red-free DMEM from Gibco (Waltham, MA, USA) supplemented with 2 mM GlutaMAX (Gibco), 10% fetal bovine serum (FBS), from Atlanta Biologicals (Lawrenceville, GA, USA), and 1 IU/mL penicillin/streptomycin (Gibco) and grown at 37 °C with 5% CO_2_. HEK293 cell lines were used to generate stable clones overexpressing the FRET-based biosensors (containing both donor and acceptor) and corresponding donor-only and acceptor-only control cell lines [2]. Three days prior to screening, the stable cell lines were expanded into T225 flasks from Corning Inc. (Corning, NY, USA). On each day of screening and FRET hit retesting, approximately 30 million cells were harvested by treatment of Tryple from Invitrogen (Carlsbad, CA, USA), washed three times in phosphate buffer solution (PBS) with no magnesium or calcium from Thermo Scientific (Waltham, MA, USA) by centrifugation at 300 g, filtered using 70 µm cell strainers (Corning), and diluted to 10^6^ cells/mL using an automated countess cell counter (Invitrogen). On each day of screening, cell viability was assessed using the trypan blue assay and found to be above 95% viable. After resuspension and dilution in PBS, the cells were continuously and gently stirred using a magnetic stir bar at room temperature, keeping the cells in suspension and evenly distributed to avoid clumping. Subcellular localization of each biosensor was evaluated using an Olympus FluoView FV1000 IX2 (Tokyo, Japan) Inverted Confocal microscope with FLIM detector. GFP and RFP signals were assessed using 473 and 561 nm laser excitation, respectively. Cell nuclei were identified by staining with DAPI, using 405 nm laser excitation.

### 2.2. Liquid Dispensing

Cells and compound mixtures were dispensed into 1536-well flat, black-bottom polypropylene plates from Greiner (Kremsmünste, Austria). Compounds were dispensed using either an automated Echo acoustic liquid dispenser from Labcyte (Sunnyvale, CA, USA) or a Mosquito liquid dispenser from TTP Labtech (Melbourn, UK). Cells were dispensed at room temperature using a Multidrop Combi liquid dispenser from Thermo (Pittsburg, PA, USA), at a density of 10^6^ cells/mL, either 30 or 120 min before screening. For screening, cells were dispensed into assay plates containing the 1280-compund LOPAC (Library of Pharmacologically Active Compounds, from Invitrogen), dissolved in DMSO. The final DMSO concentration was <1%, and control wells were prepared containing cells and matching [DMSO]. The same methods were applied for subsequent FRET testing of the reproducible hits identified in the pilot screen. Reproducible FRET hits for retesting were purchased from Tocris (Minneapolis, MN, USA) or Invitrogen (Carlsbad, CA, USA), depending on their availability.

### 2.3. FLT Detection and Data Analysis

An in-depth description of the fluorescence instrumentation has previously been described in detail and can be found in our previous publication [9]. The current instrument has been updated to a high-density microplate format (1536 wells, 5 μL/well), acquiring data from a full plate in less than three minutes with lifetime measurement precision (standard deviation/mean) of 1% or better. Fluorescence decay waveforms (with upper-end specifications of 0.2 ps resolution, 640 data points over 128 ns range) for FLT determination were detected directly as previously described [2,7], except that two different light sources were used for GFP and OFP. In both cases, fluorescence was excited with a pulsed microchip laser producing nanosecond-wide pulses. As previously described for GFP, a 473 nm laser (Bright Solutions, Via degli Artigiani, Italy) was used, delivering pulses of ~1 μJ energy at a 5 kHz repetition rate. For OFP, a 532 nm laser (Teem Photonics SNG-20F-100, Meylan, France) was used, delivering pulses of ~0.3 μJ energy at a 20 kHz repetition rate. Appropriate wavelength filters were used in each case to selectively detect the donor emission; for GFP a 517/20 bandpass emission filter, and for OFP a 586/20 bandpass emission filter. These filters were selected to exclude acceptor fluorescence, as only the lifetime of the donor fluorescent protein is required to calculate FRET (Equation (1)). The photomultiplier tube (PMT) voltage was adjusted so that the peak signals of the instrument response function (IRF) and FRET biosensor were similar. The observed time-resolved fluorescence waveform was analyzed as described previously [2], assuming a single-exponential decay, to determine the fluorescence lifetimes (FLT) for control samples containing donor only (*τ_D_*) and FRET biosensor samples containing donor plus acceptor (*τ_DA_*). FRET efficiency was calculated from

(1) $$FRET=1-\left(\tau_{DA}/\tau_{D}\right)$$

The fluorescence lifetime (mean ± SEM) from a single-exponential fit of the donor-only controls was 2.58 ± 0.02 ns for GFP and 3.55 ± 0.02 ns for OFP, in agreement with previously reported values [18,21].

For spectral detection, the data were acquired and analyzed, as described previously, to determine the spectral contributions from donor, acceptor, cell autofluorescence, and water Raman, and a similarity index (*SI*) was calculated to flag false positives due to interference from fluorescent compounds, as described previously [10]. Briefly, the high-resolution fluorescence spectra from 256 control wells containing %vol/vol DMSO were averaged and then compared to each of the 1240 wells containing the test compounds from the LOPAC small-molecule library. The shape of the spectra from the wells containing test compounds that were found to be significantly different from the DMSO control wells, as assessed by the similarity index, were flagged as false-positives.

To evaluate HTS data, the quality factor *Z*′ was determined by assessing the precision (standard deviation or SD) and FRET dynamic range [22], using DMSO as the negative control and 200 nM thapsigargin (*Tg*) as the positive control:

(2) $$Z^{\prime }=1-\frac{3\times \left(SD_{Tg}+SD_{DMSO}\right)}{\left|Mean\;FRET_{Tg}-Mean\;FRET_{DMSO}\right|}$$

### 2.4. Enzymatic SERCA Activity Assays of FRET Hits

Functional assays were performed using sarcoplasmic reticulum vesicles prepared from rabbit skeletal muscle (SERCA1a) and pig cardiac muscle (SERCA2a) [2]. An enzyme-coupled, NADH-linked ATPase assay was used to measure SERCA ATPase activity in 96-well microplates. Each well contained 50 mM MOPS (pH 7.0), 100 mM KCl, 5 mM MgCl_2_, 1 mM EGTA, 0.2 mM NADH, 1 mM phosphoenol pyruvate, 10 IU/mL of pyruvate kinase, 10 IU/mL of lactate dehydrogenase, 1 µM of the calcium ionophore A23187 from Sigma (St. Louis, MO, USA), and CaCl_2_ added to set free [Ca^2+^] to 10 μM [23]; 4 μg/mL of SR vesicle, calcium, compound, and assay mix were incubated for 20 min. The assay was started upon the addition of ATP, at a final concentration of 5 mM (total volume to 200 μL), and the rate of decrease in NADH absorbance at 340 nm was determined using a SpectraMax Plus microplate spectrophotometer (Molecular Devices, Sunnyvale, CA, USA).

## 3. Results

### 3.1. Red-Shifted FRET Pairs

The DWR method uses high-energy pulsed lasers in order to acquire thousands of photons emitted from approximately 5000 living cells expressing FRET biosensors in each 5 μL well. We investigated the fluorescence characteristics of several currently available fluorescent proteins in the appropriate wavelength ranges, to find potential red-shifted donor and acceptor FPs with the ideal properties for fluorescence lifetime HTS assays. Many factors influence whether a particular FRET pair is suitable for FLT measurements. We sought donor FPs with long lifetimes (>4 ns) to increase biosensor dynamic range. We further limited our search to FPs that can be excited using an extremely stable and fast (20 kHz) commercially available 532 nm pulsed laser. After identifying a handful of candidates (Figure A1), we examined the properties of these FPs to find FRET pairs with maximal overlap between the donor emission and acceptor absorption spectra, indicating sensitivity to detection of longer distances due to their increased Förster distance (R_0_). We also sought donor FPs with high quantum yields and acceptors with larger extinction coefficients, both contributing to increased signal intensity.

After studying several options, we selected two FRET pairs with orange fluorescent donors and a far-red acceptor. The OFP donors were mKO2 and mCyRFP1 and the acceptor mMaroon1 (MFP). The Förster distance (R_0_) was determined as described in Appendix A (Equation (A1)). We found that the mKO2-mMaroon1 and mCyRFP1-mMaroon1 FRET pairs have R_0_ values of 6.3 nm, longer than our previously reported FLT FRET pair eGFP-tagRFP (5.8 nm). Figure 1A,B depict the fluorescence excitation and emission spectra of the eGFP-tagRFP (G/R) and mCyRFP1-mMaroon1 (O/M) FRET pairs, with the area of overlap (J) between the donor emission and acceptor excitation found to be larger for the O/M FRET pair. Notably, this OFP donor can be excited using either a 473 or 532 nm pulsed laser source, although less efficiently with the shorter wavelength excitation. Our primary interest is to utilize the longer wavelength excitation to decrease fluorescence interference for HTS assays, but the possibility to multiplex GFP and OFP-based FLT measurements from a single excitation wavelength, as was done for the first FLIM experiments with the O/M pair [18], is of interest for future studies involving multiplexed HTS assays.

Fluorescent fusion protein constructs engineered with the O/M FRET pairs were generated using standard molecular biology cloning strategies. These include null linker constructs with a flexible peptide between the FPs and a FRET biosensor that monitors the structural changes of the human cardiac calcium pump (SERCA2a), as it pumps and stores calcium ions in the sarcoplasm reticulum, as required for proper heart relaxation. This two-color SERCA (2CS) [20] biosensor has been extensively evaluated using multiple FRET techniques [1,2,19,24] including single-molecule FRET [15]. It serves as a reliable FRET biosensor to evaluate our new FRET technology and assays. FRET monitors the structural status of SERCA, as affected by binding of small molecules (Figure 1C). We have found a strong correlation between SERCA function and structure, as perturbed by small-molecules, discovered through our HTS efforts in living cells [2,9]. In the present study, both human 2CS constructs (G/R and O/M) were stably expressed in HEK293 cells [10]. Confocal microscopy was performed to isolate stable clones with proper localization of 2CS biosensor at the ER membrane, as shown by the reticulated pattern surrounding the nucleus in Figure 1D. Multiple 2CS constructs with alternate FPs were also evaluated but did not increase FRET efficiency or improve the dynamic range of the biosensors. The mKO2 FRET pair was found to be unsuitable for this biosensor due to its propensity to form aggregates at the ER membrane [12]. Therefore, in the present study, we focus on the O/M FRET donor/acceptor pair mCyRFP/mMaroon.

### 3.2. Evaluation of FLT Detection Using 532 nm Pulsed-Laser Excitation

**Figure 2:**
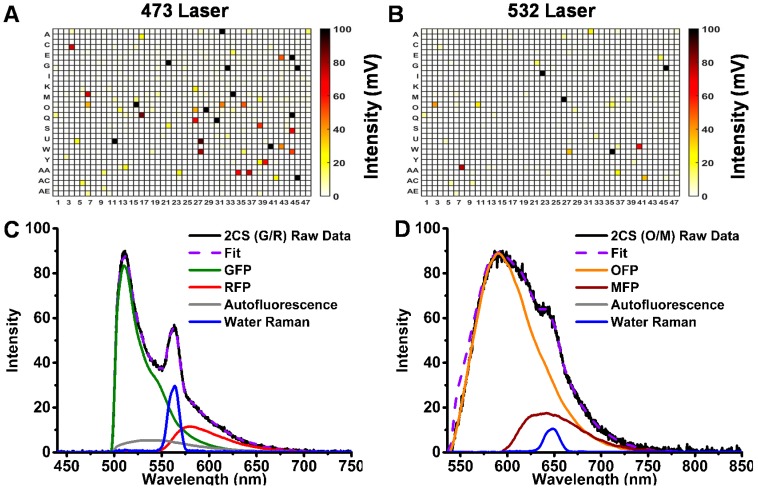
Intrinsic fluorescence interference of the Library of Pharmacologically Active Compounds (LOPAC) small-molecule library assessed in 1536-well-plate format by (**A**) 473-nm microchip laser excitation and (**B**) 532-nm laser excitation. Spectral recording analysis of stable cell line expressing (**C**) 2CS (GFP/RFP) with 473-nm excitation and (**D**) 2CS (OFP/MFP) with 532-nm excitation.

The major complicating factors in optimizing fluorescence-based HTS assays are (a) fluorescence interference from compounds that are being screened and (b) autofluorescence emitted from cells. To compare the quality of information obtained from the G/R and O/M biosensors, we screened the LOPAC library, using the 473- and 532-nm laser sources to excite the donor FPs. To screen out interfering fluorescent compounds in the LOPAC library, unlabeled HEK293 cells (no XFP expression) were screened. Interference was evaluated from the peak intensity of the FLT waveform, comparing control DMSO wells and small-molecule test wells. A fluorescent compound (false-positive hit) was flagged if the peak intensity differed (by 10% or more) from the median value observed from the 256 DMSO control wells on each 1536-well microplate. There were many more (48) interfering fluorescent compounds using 473-nm excitation than using 532-nm excitation (15), as indicated by the 1536-well plate heat maps of the LOPAC screens in Figure 2A,B.

Emission spectra (Figure 2C,D) were recorded using our recently developed spectral plate reader [10]. OFP and MFP reference spectra were generated using untagged FP constructs transiently expressed in HEK293 cells with 532-nm laser excitation. These spectra were used to resolve spectral components for the biosensors. Linear least-squares minimization, with the appropriate reference spectra, was performed and showed well-resolved fits with flat residuals across the spectral wavelengths and low χ^2^ values. Both autofluorescence (from HEK293 cells) and inelastic light scatter (water Raman) are much lower when excited at 532 nm than at 473 nm, in comparison with the donor fluorescence signal of the corresponding biosensor (Figure 2C,D). In fact, no autofluorescence signal was observed from the OFP/MFP FRET pair. Although the emission spectra of OFP and MFP are well resolved, the intensity of the MFP acceptor signal is quite low due to its low quantum yield, decreasing the potential of this particular FRET pair for screening using the spectral readout. Thus the primary role of spectral recording for this OFP-based FRET pair was to identify fluorescent compounds as false positives.

### 3.3. FLT Biosensor Dynamic Range and HTS Assay Quality (Z′)

**Figure 3:**
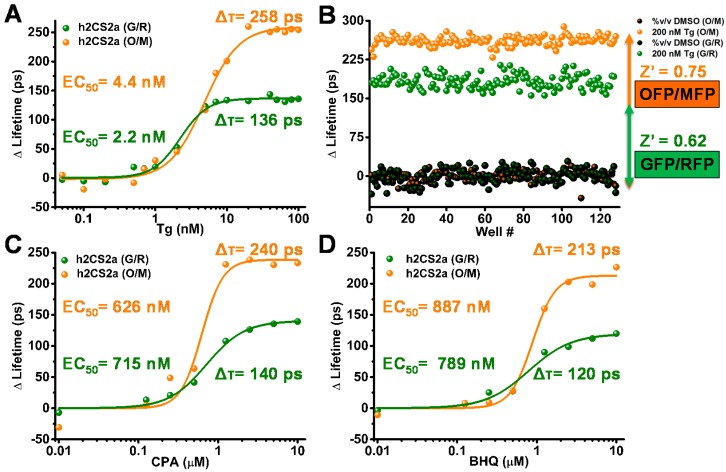
Concentration-response curves (CRC) and Z′ comparison of known small-molecule SERCA effectors. (**A**) 16-point concentration-response curves show that thapsigargin, a specific SERCA inhibitor, induces a larger lifetime change for O/M (orange) than for G/R (green). (**B**) Z′ analysis of 2CS incubated with saturating dose of 200 nM *Tg* from 128 wells of a 1536-well-plate shows a larger response for O/M than for G/R. (**C**,**D**) 8-point CRC shows that SERCA inhibitors cyclopiazonic acid (CPA) and 2,5-di-t-butyl-1,4-benzohydroquinone (BHQ) also induce larger lifetime changes for O/M than for G/R.

Concentration response curves (CRC) were generated to compare the human 2CS G/R and O/M constructs (Figure 3). Lifetime changes were tested in a 16-point concentration curve in a 384-well plate format with 50 µL sample volume per well of the well-known sub-nanomolar SERCA inhibitor thapsigargin (*Tg*). The G/R and O/M constructs showed similar EC_50_ values (4.4 ± 1.2 nM and 2.2 ± 1.3 nM, respectively). The lifetime change at saturating *Tg* was 258 ± 12 ps (mean ± SD) for the O/M biosensor, substantially greater than the value observed for G/R (136 ± 10 ps) (Figure 3A). The 12 and 10 ps standard deviations were determined from the change in lifetime. This increased “signal window” (denominator in the right term of (Equation (2)) gives the O/M biosensor substantially increased assay quality, as measured by Z′ (Figure 3B). Z′ increases from 0.62 (G/R) to 0.75 (O/M) (Figure 3B), where a value of 0.5 or greater indicates excellent precision and dynamic range for HTS assays [22]. Two factors contribute to the increased signal window: (a) R_0_ (Förster radius) is approximately 0.6 nm (10%) greater for O/M and (b) the donor lifetime for OFP in the absence of FRET (DMSO control) is about one third-greater than that of GFP.

To further evaluate the difference in the fluorescence lifetime and observed FRET response from both biosensors to other known SERCA inhibitors, 8-point concentration-response curve (CRC) experiments were performed with two other well-known SERCA inhibitors cyclopiazonic acid (CPA) and 2,5-di-t-butyl-1,4-benzohydroquinone (BHQ) (Figure 3C,D). As in the case of *Tg*, the O/M pair gave a larger lifetime change, while EC_50_ values were similar for the two biosensors.

### 3.4. Pilot Small-Molecule Library HTS Comparison

**Figure 4:**
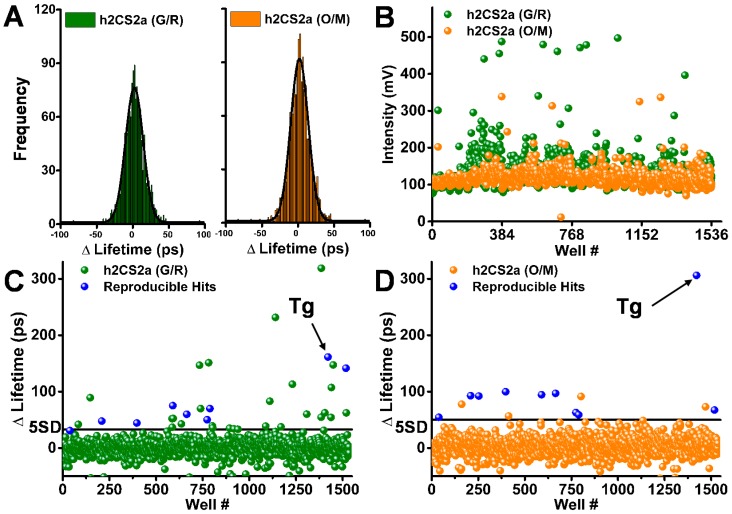
LOPAC library pilot screens for 2-color SERCA2a in live cells: (**A**) Histograms show distribution of observed lifetime changes from single screens, each fit to a Gaussian distribution to determine the SD (~8 ps for G/R and 11 ps for O/M). (**B**) Peak intensity (mV) for false-positive hit flagging. Bottom: lifetime change in a single 1536-well screen for G/R (**C**) and O/M (**D**). Known SERCA inhibitor thapsigargin (10 μM) shown by arrow. Reproducible hits (blue) were identified in three separate screens.

The two live-cell biosensors were used to screen the LOPAC library, with results illustrated in Figure 4: one-thousand-two-hundred-and-eighty compounds with a final assay concentration of 10 μM were formatted across one 1536-well plate. The remaining wells that did not contain test compounds were filled with the equivalent volume of DMSO. Stable HEK cells expressing the 2CS biosensors were harvested and diluted to one million cells per milliliter, on each day of screening. Five microliters of the diluted cells were dispensed into each well of a 1536-well plate containing all 1280 compounds from the LOPAC library. Triplicate pilot screens were performed on separate days for each biosensor. The fluorescence decay waveform from each well was analyzed to determine the single-exponential lifetime. The precision of each set of screening data were then assessed by fitting these lifetimes to a Gaussian distribution as shown in the histograms in (Figure 4A), which was used to determine the average FRET change and standard deviation (SD) for each screen. The Gaussian distribution screen analysis indicates comparable precision for the G/R and O/M biosensors, with SDs of 8 ± 2 ps and 11 ± 3 ps, respectively. Another parameter to monitor HTS precision in terms of data quality is the coefficient of variation (SD/mean) {Cornea, 2012 #106}, which was found to be less than 0.3% for both biosensors across the set of triplicate screens, indicating extremely high precision.

Spectral data of each 1536-well plate was acquired in parallel with the lifetime data, in order to evaluate the potential false-positive hits. Approximately 5% of the LOPAC compounds exhibited potential fluorescence interference. The 2CS (O/M) biosensor was developed specifically for optimizing the lifetime HTS assays and a simple analysis of the peak intensity from the lifetime waveforms for 2CS (G/R) and (O/M) depicts at least a three-fold reduction in potential fluorescent compounds from the red-shifted 2CS (O/M) biosensor. Figure 4B shows that there are more fluorescent compounds flagged by the 2CS (G/R) biosensor when screened using 473 nm pulsed excitation as compared to the 2CS (O/M) biosensor excited using 532 nm pulsed laser excitation.

Compounds that significantly and reproducibly altered the lifetime from triplicate screens of either the 2CS (G/R) (Figure 4C) or 2CS (O/M) (Figure 4D) biosensors are shown as blue in the scatter plots from one 1536-well pilot screen. Ten compounds were found to be triplicate hits from the 2CS (O/M) biosensor based on a 5 SD threshold after removing potential false positives. Compounds found as hits in all three screens for each biosensor, and also not flagged as fluorescent compounds in any one screen, were classified as reproducible hits. Notably, nine out the ten compounds were identified using both versions of the 2CS biosensor. Further, the scatter plots of all 1536 wells from one screen show that there is a significant reduction in the number of nonreproducible hits found from the 2CS (O/M) biosensor in comparison to the 2CS (G/R) biosensor, even though the precision is slightly higher for the 2CS (G/R) biosensor. For simplicity, compounds that increased the lifetime and decreased FRET are shown as hits that increased FRET were not found to be reproducible across triplicate screens. The lifetime change found from reproducible hits detected by the 2CS (O/M) biosensor were found to exhibit a larger FRET change, which is shown by response of the known SERCA inhibitor thapsigargin (*Tg*). The 2CS (O/M) biosensor identified less hits overall than the (G/R) version due to increased dynamic range and reduced interference from false-positive hits.

### 3.5. Structural (FRET) and Functional (ATPase Activity) Relationship of LOPAC Hits

**Figure 5:**
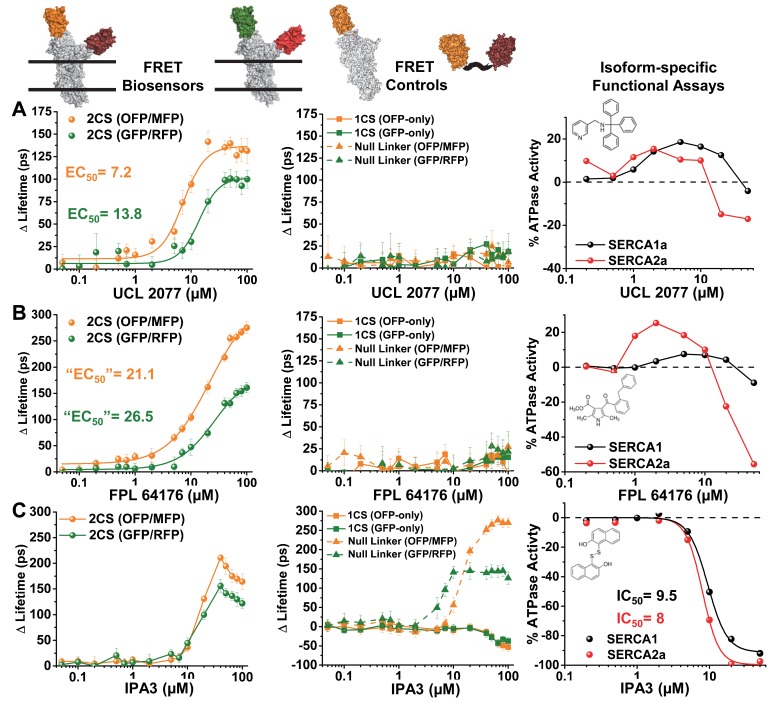
Structural and functional assays of reproducible LOPAC hits for human SERCA2a, showing concentration-response curves (CRC): (**A**) UCL 2077, (**B**) FPL 64176, and (**C**) IPA3. The first two columns show FRET-based lifetime changes for the 2CS biosensor (left) or the donor-only 1CS and null linker control cell lines (middle). The right column shows functional data (SERCA Ca-ATPase activity). The dashed line indicates the baseline ATPase activity without addition of compound. Compound structures are shown as insets.

The ten reproducible compounds that were selected as hits from the LOPAC pilot screen at 10 μM concentration were further characterized by concentration-response studies (Figure 5). These putative SERCA effectors were tested using 16-point concentration curves for the structural FRET assays using both 2CS biosensors, as well as two different sets of control biosensors. The control cell lines were utilized to determine whether or not the compounds were interacting with or affecting the fluorescent fusion proteins themselves. These control biosensors included donor-only controls and donor-acceptor “null” linker constructs, stably expressed in HEK293 cells. Ideally, it is expected that a Hit compound should produce a dose-dependent FRET change for each of the two 2CS FRET biosensors, but not for the control constructs. ATPase activity assays using two different purified SERCA isoforms were also conducted to evaluate the functional consequences of structural perturbations.

The 10 reproducible LOPAC hits produced concentration-dependent FRET changes as shown by the change in lifetime (three examples shown in Figure 5). These same 10 hits further altered SERCA’s ATPase function, with the majority found to be inhibitors. In virtually all cases, the O/M FRET pair showed a larger FRET response than the G/R FRET pair. Three different classes of FRET effectors were found to alter 2CS FRET, as illustrated by the right panel of Figure 5. Similar FRET concentration-response curves (CRCs) will be valuable for large-scale HTS and medicinal chemistry efforts on lead compounds to classify chemotypes based on structural information.

The concentration-dependent FRET responses of both the (G/R) and (O/M) 2CS biosensors, after 30-min incubation with the LOPAC hit UCL 2077, a slow after-hyperpolarization (sAHP) channel blocker [25], is illustrated in Figure 5A (right panel). This compound produced a well-resolved sigmoidal fit from the lifetime change, where a larger overall change in the lifetime was found for the (O/M) biosensor. The EC_50_ values determined from the Hill fits were in good agreement from both versions of the 2CS biosensors, where 2CS (G/R) exhibited an EC_50_ of 13.8 μM and 2CS (O/M) exhibited an EC_50_ of 7.2 μM. The corresponding donor-only and null-linker controls (middle panel) showed no significant effect on FRET, indicating that there are no off-target, nonspecific effects due to UCL2077 interacting with, or altering the properties of, the fluorescent proteins themselves. UCL2077 affected the Ca-dependent ATPase activity of two different isoforms of purified SERCA enzyme. The effect was biphasic: both isoforms were activated at low compound concentrations, but the activity peaked around 3 micromolar, then declined.

A second class of hits is illustrated in Figure 5B, where the compound FPL 64176 produced a sigmodial-shaped Hill fit across concentrations. The decrease in FRET did not saturate at the highest concentrations of both versions of the 2CS biosensors (left column), and the apparent EC_50_’s of both were nearly identical at 21 ± 3 and 26 ± 4 μM for the (O/M) and (G/R) biosensors, respectively. FPL 64176 is also a potent activator of L-type Ca^2+^ channels [26]. As in the above case, this compound had no significant effect on FRET for the control biosensors (middle panel). Interestingly, FPL 64176 produced a similar biphasic ATPase activation as UCL2077, except that it was significantly more effective (both in terms of EC50 and % change) on the cardiac isoform SERCA2a in comparison to the SERCA1a isoform found in skeletal muscle. Since FPL 64176 is known to alter the calcium dynamics of the heart, it is likely that some of its affects are due to its interaction with SERCA2a. The structural insights gleaned from the lifetime FRET HTS assays may prove important for identifying novel isoform-specific SERCA activators.

**Figure A2:**
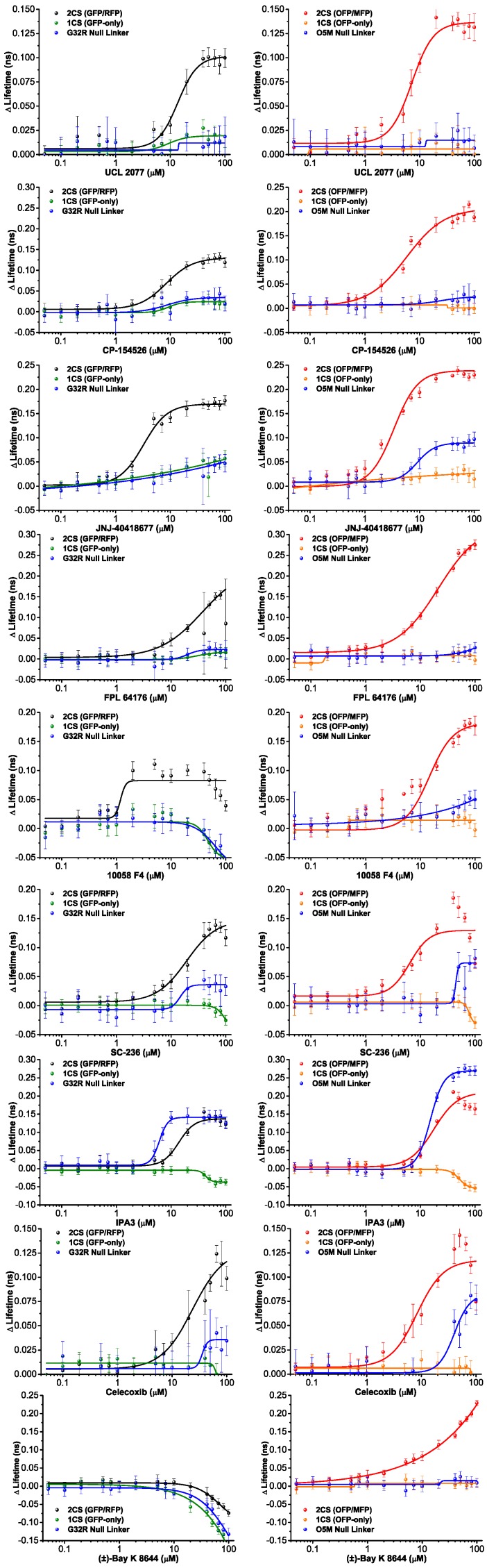
LOPAC hit concentration-response curves (CRC). Concentration-dependent FRET changes are plotted for all nine overlapping 2CS FRET hits. The left panel shows the Δ lifetime CRCs from 2CS (G/R) FRET biosensor (**black**) and the control cell lines from the GFP-only (**green**) and GFP/RFP G32R null linker (**blue**). The right side of the figure panel shows the Δ lifetime CRCs from 2CS (O/M) FRET biosensor (**red**) and the control cell lines from the GFP-only (**orange**) and OFP/MFP O5M null linker (**blue**).

The last type of FRET profile found from the reproducible 2CS FRET hits is demonstrated in Figure 5C. The compound IPA-3 was found to abruptly and significantly decrease FRET of both 2CS biosensors at approximately 10 μM. At concentrations above 20 μM the lifetime change showed an increase in FRET back toward the baseline FRET determined from the baseline controls. The EC50 values were not shown for IPA-3 because the concentration-response curve (CRC) did not have a sigmoidal trend. The fluorescent fusion protein controls for both the (G/R) and (O/M) biosensors displayed significant concentration-dependent changes in fluorescence lifetime. Specifically, both sets of the one-color SERCA donor-only controls showed even larger increases in the lifetime than found from the FRET biosensor. The null-linker constructs displayed a lifetime change in the opposite direction at higher concentrations than found for the donor-only controls. This data suggests that IPA-3 alters FRET in a nonspecific manner, potentially through its sulfhydryl moiety, which is known to form reversible covalent bonds with PAK1 and allosterically inhibit function. These redox-based effects have made IPA3 undruggable and halted clinical development of the drug [27]. IPA3 was found to inhibit the ATPase activity of the two isoforms of SERCA tested with apparent EC_50_ values found to be 9.5 μM (SERCA1a) and 8 μM (SERCA2a) in the left panel of Figure 5C. The FRET CRCs from all nine LOPAC FRET hits can be found in Figure A2. This type of structure–function profile demonstrates that compounds that interact with the SERCA enzyme in an indirect manner can be detected by the 2CS FRET biosensor, and information on the mechanism of action can be elucidated early on in the drug discovery process, potentially saving copious amounts of energy and effort.

## 4. Discussion

The focus of this study was to evaluate the OFP/MFP (O/M) FRET pair, comparing its utility with that of the more widely used GFP/RFP (G/R) FRET pair. The results demonstrate a remarkable array of advantages for the red-shifted biosensor. Our primary motivation in moving to longer wavelengths was to minimize the interference from fluorescent compounds and cellular autofluorescence, and this was clearly demonstrated in (Figure 2), resulting in a factor of three decrease in false positives. A more surprising advantage of the red-shifted pair is that the longer lifetime of OFP and the longer R_0_ further increased the sensitivity from our newly developed and refined HTS technology and FRET biosensor assays. Furthermore, the increased signal window of the red-shifted biosensors substantially improved the Z′ of our already excellent HTS lifetime-based assays (Figure 3). This increase may not appear to dramatically alter the results found from these small-scale pilot screens, but when tested on a screen of millions of compounds, this small increase in the robustness of the assay may be entirely necessary to quickly eliminate compounds having nonspecific interactions with the SERCA enzyme. It may also be necessary for quickly triaging the most promising lead compounds for further functional testing and subsequent testing in animal models of heart disease.

The utilization of a red-shifted FRET biosensor is advantageous for our DWR lifetime-based HTS technology, because more stable and reliable microchip pulsed lasers with excitation greater than 530 nm wavelengths are currently commercially available. Specifically, this work establishes feasibility for improved HTS assays targeting SERCA2a, for discovery of compounds specific for cardiac tissue and function. These screens may lead to the discovery of highly sought after small-molecule SERCA activators [28]. More generally, compounds with improved binding affinity or specificity for specific SERCA isoforms hold high potential as therapeutics for the treatment of heart failure, type II diabetes, and numerous other degenerative diseases. Even more generally, this approach is likely to have wide application to any identified protein target for which the engineering of FP biosensors is feasible.

## 5. Conclusions

We have described red-shifted fluorescence resonance energy transfer (FRET) biosensors, targeting the cardiac calcium pump, that significantly improved dynamic range, signal-to-noise, and hit selection for high-throughput screening (HTS). When coupled with a newly-updated HTS microplate reader, these red-shifted FRET biosensors enable unprecedented precision and assay quality in nanosecond fluorescence decay (lifetime) measurements from microliter sample volumes at three minutes per 1536-well plate. This combination promises to revolutionize high-precision FRET measurements from living-cells for the discovery of urgently needed therapeutics.

## Appendix A

FRET occurs by the transfer of non-radiative energy between dipole-dipole interactions, typically between a shorter wavelength donor molecule, in the excited electronic state, to a longer wavelength acceptor molecule in the ground state. The probability that energy transfer will occur depends on the spectral overlap between the emission and absorption spectra of the donor and acceptor, the fluorescence quantum yield of donor, the molar absorptivity (extinction coefficient) of the acceptor, and the distance and orientation of the dipole interaction. The probability of energy transfer is defined as FRET efficiency (*E*), which is directly proportional to the rate of energy transfer (*k*_T_) and the R^−6^ distance dependence of the donor and acceptor. The Forster distance (*R*_0_) shown in (Equation (A1)) is the distance between a particular donor-acceptor FRET pair, at which the probability of FRET efficiency is 50%.

(A1) R0=9,790[J(λ)κ2η−4φD]16

The Forster distance (*R*_0_) is defined by the area of overlap between the donor emission and acceptor excitation fluorescence spectra *J*(*λ*), the relative orientation of the transition dipoles of the donor and acceptor *κ*^2^, the quantum yield of the donor *φ_D_* (not in the presence of the acceptor), and the refractive index of the medium *η*. The orientation factor (*κ*) is usually assumed to be equal to 2/3. This is appropriate for donor and acceptor pairs attached by flexible linkers, where motion is dynamic, and not sterically restricted. *R*_0_ is most affected by the overlap integral *J*(*λ*) of a particular FRET pair. The ability to determine the distance between two fluorescent probes allows FRET to be used as a powerful tool to determine molecular interactions, and directly quantitate the measurement in terms of distance and possibly orientation. The sensitivity of FRET is determined by the Forster distance (*R*_0_), and is highest when the distance between the donor and acceptor probes is near *R*_0_.
